# The unstable shoulder: what soft tissue, bony anatomy and biomechanics can teach us

**DOI:** 10.1007/s00167-021-06743-0

**Published:** 2021-09-24

**Authors:** Lukas N. Muench, Andreas B. Imhoff

**Affiliations:** Department of Orthopedic Sports Medicine, Klinikum Rechts Der Isar, Technical University of Munich, Ismaninger Str. 22, 81675 Munich, Germany


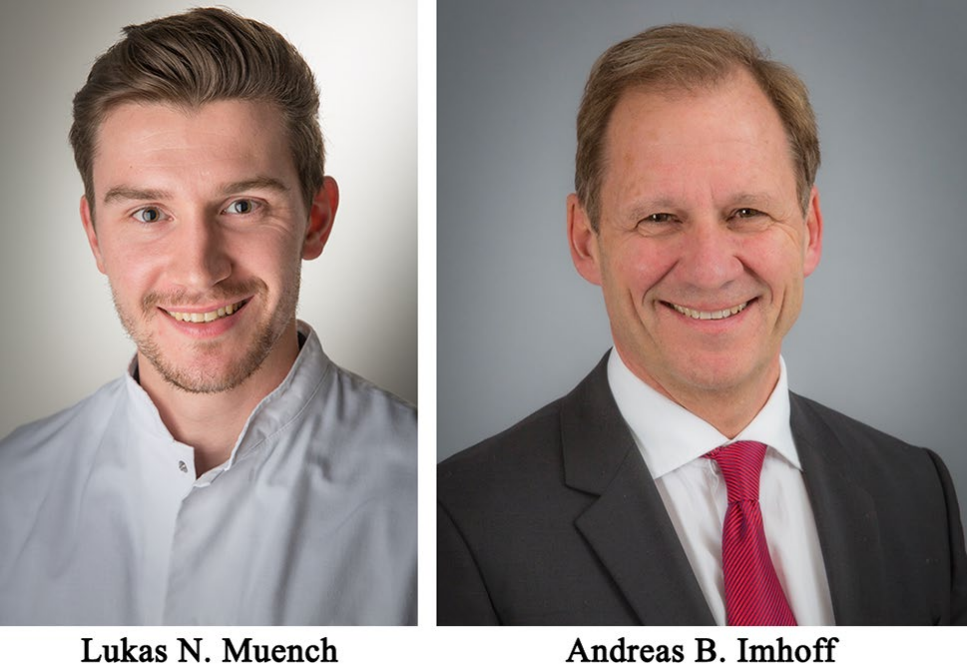
Even though shoulder instability has been extensively studied, therapeutic approaches for certain patient subgroups remain a matter of ongoing debate. This is mainly due to the existing controversy on how to address individual patient-related risk factors, including the variability in soft tissue properties and bony anatomy, to prevent recurrence of instability.

In general, high-level evidence suggests that young, physically active patients should undergo surgical stabilization after first-time traumatic anterior dislocation, due to the alarmingly high rate of recurrence [10]. Repair of the anteroinferior capsulolabral complex is usually performed arthroscopically using a minimum of three suture anchors, ensuring an anatomic soft tissue restoration, sufficient biomechanical stability, and satisfactory functional outcomes [1, 5, 12]. Recently, the importance of adequate placement of the most inferior anchor at the 5:30 o’clock position and the height of the created glenoid labral “bumper” at this specific position has been highlighted for reducing the rate of recurrent instability and maximizing postoperative success [13]. It is also worth mentioning that a “diffusely small” labrum, defined as a labral height of less than the width of the glenoid tidemark cartilage, has been shown to be associated with the occurrence of postoperative re-instability [21]. Consequently, detailed assessment of labral and capsular morphology is critical pre- and intraoperatively, as patients with a small labrum and/or a large capsular volume will most likely benefit from performing an additional capsular shift. Accordingly, shoulder surgeons should also be aware of the re-stretching trait of the capsule along with a re-increase of capsular volume even after successful surgical interventions, potentially leading to recurrent instability [18].

Interestingly, increased capsular volume rather than ligamentous laxity per se has also been suggested to be the critical morphological feature of shoulder hyperlaxity, observed in approximately 13% of patients with first-time dislocations [7, 11]. Treatment of patients presenting with antero-inferior instability and concomitant hyperlaxity remains a major challenge, due to hyperlaxity being an independent risk factor for recurrent instability and a predictor for failure following arthroscopic Bankart repair [3, 17]. As previous studies demonstrated a reduction of capsular volume by 57% in the setting of combined anteroinferior and posteroinferior capsular plication [23], an additional suture anchor should be placed posteroinferiorly at the 7 o’clock position to create a superomedial capsular shift. Biomechanically, performing the plication stitch in an inferior-to-superior direction preserved rotational range of motion more sufficiently when compared to a medial-to-lateral direction [2].

Despite advances in surgical techniques and instrumentation, arthroscopic capsulolabral repair should not be considered the panacea for all patients suffering from shoulder instability. Especially bone loss at the anterior rim of the glenoid has been identified as the number one cause for failure following soft tissue-based shoulder stabilization. Historically, 20% to 25% has been deemed the “critical” cut-off value where glenoid bone loss should be surgically addressed using a bone augmentation procedure [8, 20]. Once again, the debate regarding a redefinition on what constitutes the correct threshold value has flared up, as a “subcritical” bone loss of 13.5% was found to significantly impair functional outcomes following arthroscopic Bankart repair, questioning if these patients would have benefitted from bone grafting [20]. The term “subcritical” implies that a bone loss of 13.5% is associated with clinically relevant worsening of functional outcomes. In contrast to the presence of “critical” bone loss, however, an increased rate of postoperative recurrent instability is not observed.

Unsurprisingly, there is not only a lack in consensus on the “critical” threshold value of glenoid bone loss, but also on which of the various bone augmentation techniques should be considered the optimal choice for these patients. Numerous techniques are currently in clinical use, including transfer of the coracoid, use of a tricortical iliac crest or scapular spine autograft, and fresh cadaveric allografts [8]. When conducting a hypothetical survey, our French neighbours would most likely advocate that each of these patients should undergo the Latarjet procedure. Undoubtedly, the Latarjet procedure—either performed open or arthroscopically—has been proven to consistently achieve sufficient functional outcomes along with low rates of recurrence [8, 24]. However, especially when performed open, the risk for neurovascular injuries as well as concerns regarding the development of glenohumeral osteoarthritis and the technically more demanding surgery in the revision setting clearly challenge the indication of a primary Latarjet procedure in cases with negligible glenoid bone loss [9]. In addition, the amount of coracoid graft osteolysis has been shown to be disturbingly high in patients with a glenoid bone loss of less than 15%, probably due to the insufficient mechanotransduction effect between humeral head and bone graft [6].

When assessing bone loss at the humeral head—better known as the Hill Sachs lesion—everyone should be familiar with the “glenoid track” concept, highlighting the importance of position and orientation of the lesion [25]. In the setting of isolated humeral bone loss, “off-track” Hill Sachs lesions, which are at risk for engaging with the glenoid and causing recurrent instability, are most frequently treated using a remplissage procedure, where the infraspinatus and posterior capsule is transferred into the defect [8]. Biomechanically, the infraspinatus tenodesis may prevent the humeral head from anterior translation, subluxation, and engagement with the glenoid [4]. However, in cases of bipolar bone loss, lengthening the glenoid arc with a bone block procedure may be preferred, effectively converting the Hill Sachs lesion from “off-track” to “on-track” without the need for an additional remplissage [15, 19].

Recently, a rethinking process involving the impact of glenoid concavity has been initiated, challenging the current concept of determining a general threshold value for critical glenoid bone loss as a criterion for the use of a bone augmentation procedure in shoulder instability [16, 22]. Ironically, previous work already demonstrated that the concave shape of the articular surface of the glenoid should be considered the crucial component for determining glenohumeral stability, which is based on the synergism with the rotator cuff via the “concavity compression” principle [14, 26]. However, the effect of glenoid concavity and its relationship to bone loss has been brought to the fore more than ever, as the same extent of glenoid bone loss in an increasingly concave-shaped glenoid was found to result in a greater decline in stability when compared to a flatter glenoid [16]. Consistently, a cadaveric study observed that the loss of stability with increasing defect size was dependent on initial concavity [22].

Consequently, simply measuring the extent of glenoid bone loss may not provide the precise answers we thirst for regarding its true biomechanical effect, as the inter-individual, biomechanically relevant differences in glenoid concavity may skew the truth. However, it remains to be seen whether the consideration of glenoid concavity will substantially influence treatment algorithms in the future and pave the way for an even more individual surgical approach.
